# The Dental and Tonsil-Adenoid Clinics

**Published:** 1920-11

**Authors:** Harvey J. Burkhart


### THE DENTAL AND TONSIL-ADENOID CLINICS

BY HARVEY J. BURKHART, D.D.S.

The tonsil-adenoid clinic conducted by the Rochester Dental Dispensary, Rochester, N. Y., July 26 to September 10, 1920, operations were performed on 1,470 children under the age of 16 years. The expenses of the clinic amounted to $14,411.19. A nominal fee of $5 per child was charged in all cases where the family was financially able to pay it, and there was received from that source

$7,082.50, leaving a deficit of $7,328.69, that was met by a special fund.

The 1,470 operations were performed without a single casualty. Every effort was made to provide proper facilities and appliances for doing the work in a cleanly, orderly and efficient manner. The children were frequently examined after operation and closely watched so that if bleeding started they were immediately given attention. Credit should be given to the operators for the extreme care exercised in performing the operations. It is the opinion of those closely associated with the work that it was due to this care that no "surgical accidents" occurred.

Concurrently with the clinic, daily examinations were held, affording parents the opportunity to have their children examined and, if found to need operation, listed for treatment. Out of a total of 2,666 children examined, 2,493 were found to be in need of treatment and were listed. While it early became apparent that the number of operable cases would be in excess of the capacity of the clinic, the examinations were continued in order to ascertain the true situation and to stimulate the provision of ways and means to meet it.

The publication of this report is prompted by requests received from organizations and individuals in various part of the country for information as to how the clinic was conducted, plan of organization, methods and machinery employed, and various details. In recent years clinics have been held in a number of rural communities by boards of health and other organizations, but so far as is known, no data was kept as to methods employed, expenses incurred and results achieved. It is hoped, therefore, that the information made available by this clinic and as herein set forth, may prove of value in the conduct of other clinics in other parts of the country. A large number of communities do not yet realize the necessity for and importance of preventive surgery, or have put off from time to time having it done, with the result that there are thousands of children suffering discomfort and denied the chance to develop mentally and physically as they should were their cases attended to. It is undoubtedly true that as a general rule the far-reaching importance of tonsil-adenoid corrective work and the great benefit that would result from it, not only to this but future generations, by giving children a chance to develop normally, is overlooked, and as a result a great injustice is done children and the community at large. It is hoped that the results of this clinic will stimulate other organizations, not only in Rochester, but elsewhere, to bear their fair share of the burden of this most important preventive and remedial work.

Organization and supervision of the surgical staff and its work were in charge of Edwin S. Ingersoll, M.D., and the work of organizing and supervising the history and medical examinations was directed by Albert D. Kaiser, M.D. The attention of the reader is particularly directed to the statements published herein by Dr. Ingersoll on the surgical phase of the clinic, Dr. Kaiser on the medical phase, and Dr. George W. Goler, health officer of the city of Rochester, on the importance of continuing the work so that the number of operable tonsil-adenoid cases may at all times be kept well in hand and at a normal level. A complete report of this experiment can be had by addressing the Rochester Dental Dispensary, Rochester, N. Y.

---

Vaseline your screw mandrels to keep them from rusting. It doesn't boil out easily.

Wax your instrument when conveying cement or pro-tem. to the cavity. It lets go of the instrument easier.

When grinding teeth place a wet piece of tissue paper on the tongue. It catches most of the debris.—H. B. Ward.

